# Comprehensive study on gear oil modified with lignin–cellulose nanofibers: rheological and tribological response

**DOI:** 10.1038/s41598-026-69723-9

**Published:** 2026-09-15

**Authors:** Galal Sherif, Ahmed Nabhan, Esraa M. Abd Elsadek, Mohamed Ali

**Affiliations:** 1 Production Engineering and Mechanical Design Department, Faculty of Engineering, Minia University, El-Minia, 61111 Egypt; 2 Mechatronics Program, Faculty of Engineering, Minia National University, El-Minia, 61519 Egypt; 3 Mechanical Engineering Department, College of Engineering and Technology, Arab Academy of Science, Technology and Maritime Transport, Sadat Road, P.O. Box 11, Aswan, Egypt; 4 Department of Mechanical Engineering, Higher Technological Institute, 10th of Ramadan City, Egypt; 5 Mechatronics Engineering Department, Faculty of Engineering, Delta University for Science and Technology, Gamasa, Egypt

**Keywords:** LCNFs, Gear oil, Lignin-cellulose, Sustainable, Dispersion stability, Engineering, Materials science

## Abstract

The tribological, rheological, and acoustic performance of gear oil dispersed with lignin-cellulose nanofibers (LCNFs) are examined. Samples were performed with loading content of 0.25%, 0.5%, 0.75%, and 1.0%. The morphological and structural characterization of the LCNFs was confirmed using AFM, SEM, XRD, and FT-IR evaluations. The results revealed a substantial reduction in COF and wear rate at best loading content, with 0.5% by weight LCNFs, by up to 42% and 25.5%, respectively. Vibration and sound pressure level measurements showed significant damping behavior, with reductions of 18–25% at low and optimal loading level. Surface analyses of worn specimens (3D topography and SEM) confirmed the formation of a protective LCNF tribo-film at optimal dispersion, while excessive loadings above 0.5% by weight LCNFs resulted in abrasive features associated with particle clustering. In summary, LCNFs seem to be a very promising and fundamental ingredient in gear oil. The evolution of highly effective and sustainable nano-lubricants is highlighted by the potential to improve dynamic response, lower wear and friction, and increase viscosity stability.

## Introduction

 The working conditions of gear lubrication systems were more intricate compared to the characteristics of engine oils^1,2^. The slipping action of gear teeth elevates contact pressures and localized temperatures, resulting in expedited wear and degradation. Thus, checking the gear oils and the inclusion of chemicals are essential to augment load-bearing capacity, promote protective film formation, and minimize scratching, wear, and vibrations^3–5^. The latest research efforts are focused on integrating nanoparticles into gear oils to enhance their rheological and tribological characteristics.

The main ingredients of lubricating systems were metal oxide and carbon nanoparticles^6,7^. Metal-oxide NPs disclose unique qualities, which include a rolling effect, forming ultra-thin films, a superior viscosity index, and inhibiting asperity-asperity interaction. Moreover, nano-carbons provide outstanding self-lubrication potential and mending of the micro pores^8,9^. It was indicated that incorporating both metals and metal-oxide nanoparticles enhances the tribological performance of hydroxypropyl methylcellulose coatings^10^. Beyond that, the hybrid fillers contribute to strengthened dispersion resistance during severe shear scenarios. Hybrid nanofillers offer numerous benefits thanks to the combination of the unique properties of each component^11–13^. The synergistic function of hybrid nanofillers effectively enhances wear resistance and improves thermal stability. TiO_2_, MoS_2_ and CaF_2_ NPs were adopted as vegetable oil additives to evaluate gearbox performance^14^. It can be observed that power loss minimizes approximately 48%, 56%, and 60% with TiO_2_, CaF_2_, and MoS_2_. TiO_2_ and SiO_2_ NPs were incorporated into a pair of industrial engine oils to upgrade the tribological function^14,15^. It was demonstrated that samples with a loading level of 1.0% by weight of TiO_2_ NPs reduced COF and wear rate by 39% to 47%, respectively. While a loading amount of 0.5% by weight of SiO_2_ NPs increased tribological characteristics. MoS₂ and ZnO NPs were added to the diesel oil (SAE-40 monograde). It was proven that MoS₂ NPs greatly reduced the pin mass loss by up to 93%. Even though both nano-additives tend to increase viscosity up to 10%^16,17^. MWCNTs and TiO_2_ NPs were introduced to turbine meter oil to explore the wear process. It was found that loading content of 0.4 wt% of MWCNTs and 0.1 wt% of TiO_2_ contributes to reducing the wear depth approximately 88% and 71%, respectively. The kinematic viscosity at 40° rises up to 22% with 0.3 wt% of both nano-additives^18^. Engine oil 10 W-30 suspended low loading amount of TiO2 NPs was performed. It was demonstrated that COF was dropped by approximately 80% with 0.075 wt% TiO2, at working temperature 75°^19^. An examination was conducted utilizing machine oil suspended with 0.1 wt% cesium-hybridized graphene oxide quantum dots (Cs-GOQDs). It was observed that the average wear scar diameter dropped by roughly 9.2% and the COF decreased by about 14.6%^20^.

Hybrid filling systems that utilize MWCNTs with various metal oxides. MWCNT/SiO_2_ integrated into SAE68 indicates a reduction in COF and scar width up to 93% and 14%, respectively^21^. Once 0.25% of hybrid ZnO/MWCNTs are added to 10W40 engine oil, COF and volume loss drop by 32% and 75%, respectively^22^. Hybrid MWCNTs/Al_2_O_3_ exhibit a decrease in COF and mass loss of 48% and 53%, respectively^23^. Hybrid nanoparticles can enhance wear resistance, promote thermal stability, and increase load-carrying capacity. However, the environmental impact of conventional nanofillers presents challenges related to their low biodegradability and short-term suspension within the lubricant^24^.

Bio-derived nanoparticles consequently emerged as sustainable potential for next-generation gear-oil enhancers^25^. Cellulose nanofibers (CNFs) and cellulose nanocrystals (CNCs) have been identified to increase the viscosity index greatly and shear resistance through network interlacing^26^. Its large surface area and dispersibility in polar liquids also facilitate its adhesion to slippery surfaces^27^. The incorporation of CNC in SAE 40 lubricant enhances its wear and friction resistance capabilities. It was indicated that nano-lubricants with 0.1% CNC contribute to decreasing the COF by 69%. The results were validated using wear trace analysis under working conditions of low speed and high load^28^. It was demonstrated that 1% CNC was suspended in SAE40 lubricant leads to raise the viscosity index by 2.53%^29^. A blend of CNC and CuO was introduced into SAE 40 base oil to verify its wear, friction, and rheological durability. It was observed that loading content of 0.3% of CNC-CuO, equal ratio, leads to increasing COF up to 40% at low loading condition^30^. The hybrid CNC-CuO with 70:30 filling ratio was adopted to evaluate the rheological and tribological attributes. It was verified that incorporating 0.5% by weight of hybrid CNC-CuO exhibits a high thermal conductivity ratio and specific heat capacity scenario. The dynamic viscosity and viscosity index increased up to 75% and 47%, respectively^31^. The COF decreased by approximately 48–50% under boundary lubrication conditions. Under mixed and hydrodynamic lubrication regimes, the COF decreased by nearly 33–44% and 9–13%, respectively. In addition, the wear rate improved by approximately 33.5%^32^. It can be evident that 1.4% of CNFs, extracted from rice traw, maximizes the viscosity index of waste oil by 15%, while scar width decreases approximately 31%^33^. It was revealed that dispersion of 2% by weight of modified CNCs in polyalphaolefin base oil contributes to a low COF of about 30%^34^. It was demonstrated that incorporating 1% by weight of CNCs in SAE40 oil contributes to a viscosity index of approximately 2.5%^29^. CNCs were adopted as a lubricant enhancer of isopropyl palmitate oil. It was indicated that the viscosity index was evaluated at about 9%, while COF was decreased by nearly 44%, with a loading content of 0.25%^13^. Hybrid CNCs-Al_2_O_3_ NPs were utilized to synthesize bio-gear oil. It was demonstrated that a loading level of 2% with an equal ratio causes the viscosity index to rise by roughly 17%. On the other hand, the wear scar and noise level drop by around 36% and 11%, respectively^35^. Ficus carica oil blended with Eichhornia crassipes carboxymethyl cellulose (EC-CMC) was performed. It was demonstrated that the COF decreased by approximately 26% with 2 wt% EC-CMC compared to the base oil, while the lowest wear scar diameter of 0.39 mm was obtained^36^.

Table 1 highlights the significant potential of bio-derived cellulose materials for lubricant applications. Previous studies mainly focused on CNFs and CNCs as lubricant additives. However, limited attention has been devoted to lignin-containing cellulose nanofibers (LCNFs). The lignin in LCNFs could stimulate interfacial interactions within the lubricating system. LCNFs may possibly increase oxidative stability and encourage the development of a persistent protective tribo-film. Therefore, LCNFs provide a promising sustainable additive for advanced gear-oil utilization scenarios.

**Table 1:** An overview of the impact of bio-derived cellulose-based lubricating oil modifiers.

Base Oil/Lubricant Type	Additive	Loading Content	Reported Improvement/Observation	Ref.
SAE40 oil	CNCs	0.1 wt%	COF reduced by ~ 69%; improved wear resistance under low-speed and high-load conditions	^28^
SAE40 oil	CNCs	1.0 wt%	Viscosity index increased by ~ 2.53%	^29^
SAE40 oil	Hybrid CNC–CuO (equal ratio)	0.3 wt%	COF increased by ~ 40% under low loading conditions	^30^
SAE40 oil	Hybrid CNC–CuO (70:30 ratio)	0.5 wt%	Dynamic viscosity increased by ~ 75%; viscosity index increased by ~ 47%; enhanced thermal conductivity and specific heat capacity	^31^
SAE40 oil	Hybrid CNC–CuO	0.5 wt%	COF reduced by ~ 48–50% (boundary), ~ 33–44% (mixed), and ~ 9–13% (hydrodynamic); wear rate improved by ~ 33.5%	^32^
spent engine oil 10W30	CNFs extracted from rice straw	1.4 wt%	Viscosity index increased by ~ 15%; wear scar width reduced by ~ 31%	^33^
Polyalphaolefin base oil	Modified CNCs	2.0 wt%	COF reduced by ~ 30%	^34^
SAE40 oil	CNCs	1.0 wt%	Viscosity index increased by ~ 2.5%	^29^
Isopropyl palmitate oil	CNCs	0.25 wt%	Viscosity index increased by ~ 9%; COF reduced by ~ 44%	^13^
Bio-gear oil	Hybrid CNCs–Al_2_O_3_ NPs	2.0 wt%	Viscosity index increased by ~ 17%; wear scar reduced by ~ 36%; noise level reduced by ~ 11%	^35^
Ficus carica oil	EC-CMC	2.0 wt%	COF decreased by approximately 26%	^36^

Consequently, this research project seeks to evaluate the influence of LCNFs distributed in gear oil on its rheological and tribological performance. In addition to evaluating the worn surfaces to clarify the fundamental wear mechanisms, we also performed viscosity behavior analysis, infrared spectrum analysis, and vibration performance.

## Materials and characterization details

The current test oil sample is gear oil, HP EP 90, that exhibits excellent thermal, rust and corrosion reaction, and physical performance under extreme pressure. The specifications of oil are dynamic viscosity of 14.5–17 cSt at 100 °C, viscosity index of 90, and flash and pour temperature points of 180 °C and − 9 °C, respectively. The added ingredient is cellulose extracted from rice straw, which maximizes the benefits of sustainability and environmental decomposition. Rice straw is widely available in Egypt, and using nanocellulose extraction contributes to the safe disposal of waste without burning. The cellulose extraction process involves several stages. The first stage utilizes a straw grinding technique at 160 °C, carried out in an aqueous solution of sodium sulfite and sodium carbonate at concentrations of 10% and 2% of the straw’s weight, respectively, for 120 min. This process synthesizes unbleached, neutral-sulfur straw pulp. The second stage involves nanofiber production using an ultra-fine friction mill (model MKZA6-2, Masuko Sangyo Co., Ltd., Kawaguchi, Japan). The extracted nanofibers are calibrated and tested to determine their properties, as shown in Table 2.

**Table 2:** Data sheet of physical properties of LCNFs.

Specifications	Value
Average size (µm^2^)	11.94 ± 6.5
Transmittance (%)	18.63 ± 2.1
Nanosized fraction (%)	78.86 ± 3.9
Density (g/cm^3^)	0.97 ± 0.21
Young’s modulus (GPa)	6.3 ± 0.2
Quality Index	81 ± 11.8

The test samples were prepared with 0.25%, 0.5%, 0.75%, and 1.0% by weight of LCNFs formulations at a room temperature of 25 °C and 54% relative humidity. The dispersion of LCNFs is a major challenge; therefore, a dissolving agent was employed to promote excellent dispersion and ensure suspension. Oleic acid was utilized as the solvent due to its surfactant features, which reduce agglomeration and facilitate optimal dispersion^37^. The nano-lubricant samples were prepared by combining LCNFs with oleic acid using a mechanical stirrer (Daihan MSH 20D - Vietnam) at a constant speed of 250 rpm for 100 min to ensure initial homogeneity. Subsequently, the nano-lubricant samples underwent ultrasonic dispersion using a cleaner device (Diaham WUC-A03 - Korea) for 15 min to reduce agglomeration. The samples were left to stand for up to 90 days after preparation to ensure the stability of the nanolubricant’s structure (see Table 3).

**Table 3:** LCNFs-gear oil sample formulations.

Labels	Gear Oil	Oleic acid	LCNFs
GO-Free	100%	-	-
GO-OA	98%	2%	-
GO-LCNF01	97.75%	2%	0.25%
GO-LCNF02	97. 5%	2%	0.5%
GO-LCNF03	97.25%	2%	0.75%
GO-LCNF04	97%	2%	1.0%

The structural properties of the nanolubricants were evaluated through viscosity measurement and spectroscopic calibration. Dynamic viscosity (DV) measurements of the nanolubricants were carried out at working temperatures of 40 °C and 100 °C. The experiments were carried out according to ASTM D446 using a rotating viscometer (B-One Plus LR - France)^38^. The viscosity index (VI) was identified by reference to ASTM D2270^39^. Infrared spectroscopy analysis (FTIR) was conducted utilizing a spectrophotometer (JSX-1000 S; JEOL - Japan), and the data were provided in the range of 500 to 4000 cm⁻¹.

An experimental vibration setting (G.U.N.T. PT500 - Germany) is used in this work to record displacement and acceleration signals based on ISO 10816-3^40,41^. The experiments were conducted under different operating conditions, where the rotation speed was 500, 1000, 1500, 2000, and 2500 rpm under a normal load of 200 N. Accelerometers (IMI Sensors-603C01) are installed on a tested bearing housing to measure acceleration signals in two directions (vertical and horizontal)^42^. A data acquisition card (BMC USB-AD16F) is used to collect signals. A sound level equipment (TOTAL, Test01 - China) carried out to evaluate the noise levels in decibels (dB), by refereeing ASTM C423^43,44^.

Experiments using a block-on-ring tribometer calibrated the COF and mass loss (∆m), according to ASTM G77-17 standards^45^. The samples were checked five times to verify accuracy and limit reading errors. For every oil sample, a virgin aluminum plate was utilized, and surfaces were cleaned with acetone. Moreover, the wear mechanism was performed under sliding distance settings. The wear rate was estimated by tracking mass loss.

1 $$\:W=\frac{{V}_{disc}}{{F}_{n}\:*S}$$

2 $$\:{\mathrm{V}}_{\mathrm{d}\mathrm{i}\mathrm{s}\mathrm{c}}=\frac{\varDelta\:\mathrm{m}}{\rho\:}\:\:\times\:1000$$

Consider the volume loss $\:{V}_{disc}$ (mm³), mass loss $\:\varDelta\:\mathrm{m}$ (g), slide density $\:\rho\:$ (g/cm^3^), and applied load $\:{F}_{n}$ (N).

The topography of sliding surfaces is examined to understand the wear mechanism. An optical microscope (OLYMPUS BX53M - Japan) is used to image wear traces and slip marks. A scanning electron microscope (SEM; JSM-IT200; JEOL - Japan) is used to observe the damaged areas with high precision. The examinations help determine the nature of the damage and analyze the microstructure.

## Results and discussions

### LCNFs characterizations

**Fig. 1:**
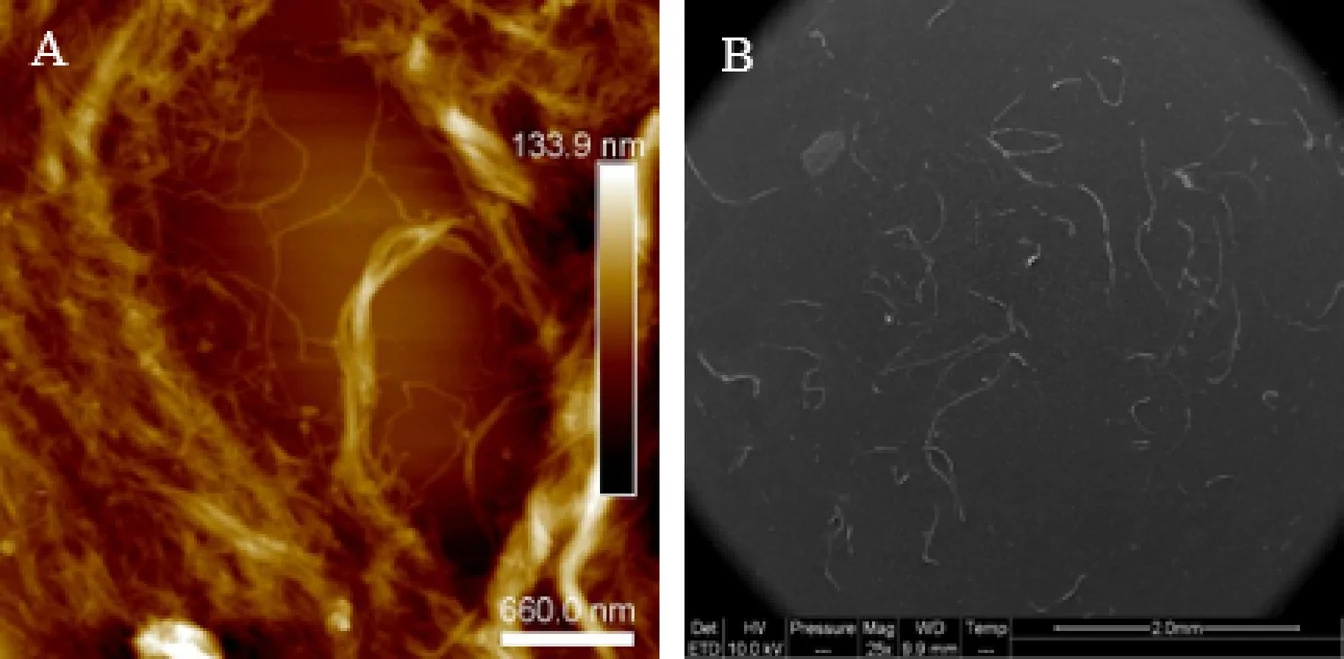
**(a)** AFM and **(b)** SEM of LCNFs.

Fibers are examined to assess the structure and arrangement following cellulose extraction. A topographic view of the LCNF fibers, showing the shape and distribution of the fibers, is displayed in Fig. 1a. The bright areas in the AFM image correspond to the upper regions of the fiber surface, indicating the lattice structure of the interwoven lignin fibers. SEM scan exhibits a detailed visualization of elongated, interwoven fibrous structures, indicating the compatibility of the cellulose and lignin components, as demonstrated in Fig. 1b. This can confirm the successful extraction of nanofibers with uniform dispersion and nanoscale dimensions^46^.

**Fig. 2:**
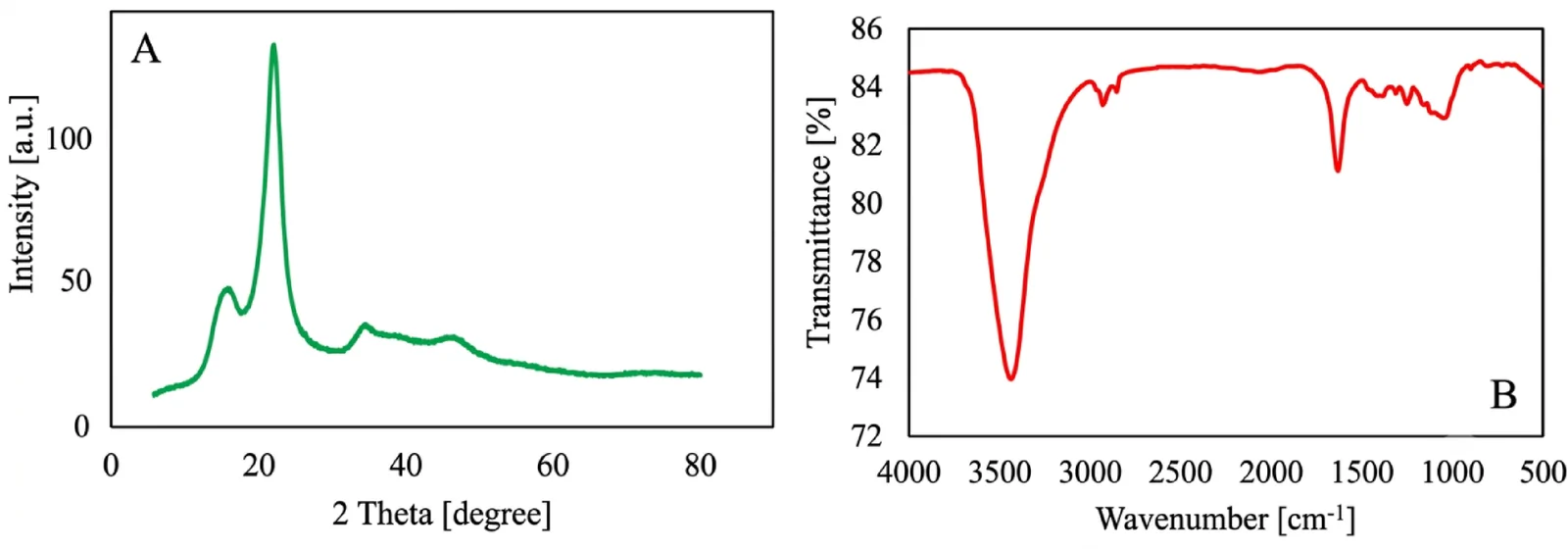
**(a)** XRD and **(b)** FTIR of LCNFs.

The X-ray diffraction (XRD) pattern of the crystalline phases of lignin-cellulose nanofibers was demonstrated in Fig. 2a. Characteristic peaks can be observed at specific 2θ values around 16° and 22°. Moreover, the widespread convexity phenomena can indicate the presence of amorphous lignin areas^47,48^. It can be confirmed that the presence of crystalline cellulose and amorphous lignin phases contributes to the balance between mechanical strength and elasticity in the nanofibers. The infrared spectroscopy (FTIR) is conducted to characterize the functional groups present in chlorinated LCNFs. It can be revealed that the peak at 1050 cm^− 1^ can describe the glycosidic bonds in cellulose (C–O–C stretched). Furthermore, peaks at 1600 cm^− 1^ (aromatic ring vibrations) and 1730 cm^− 1^ (C = O stretched) may be typical of lignin and lignin ester bonds, respectively. A peak at 2900 cm^− 1^ illustrates the growth of aliphatic structures (C–H stretched). Furthermore, the presence of hydroxyl groups linked by hydrogen bonds in cellulose (O–H stretching) is proved via a crest at 3330 cm^− 1^. It can be summarized that the functional interphase of LCNFs confirms the formation of lignin and cellulose and their chemical bonding within the matrix^47,49^.

### Structure and rheological properties of nanolubricants

**Fig. 3:**
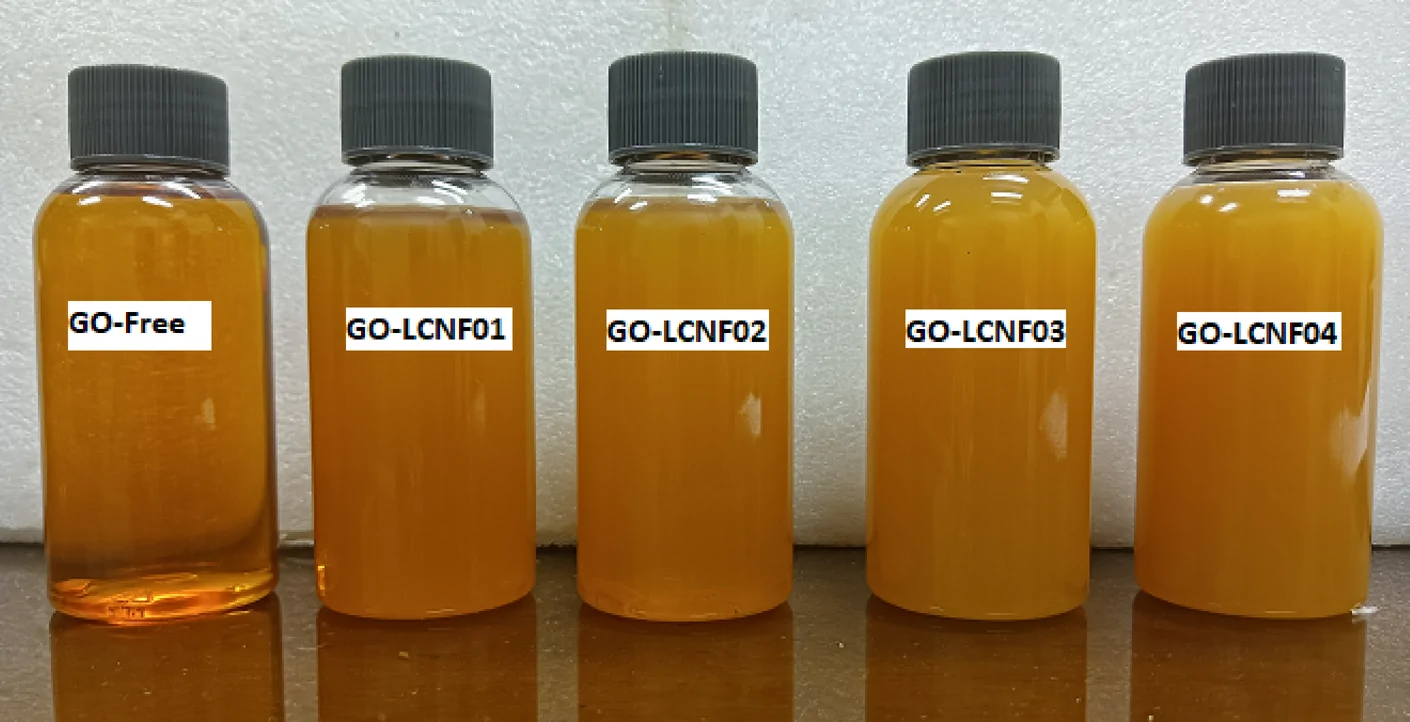
Sedimentation of gear oil dispersed with LCNFs after three months.

**Fig. 4:**
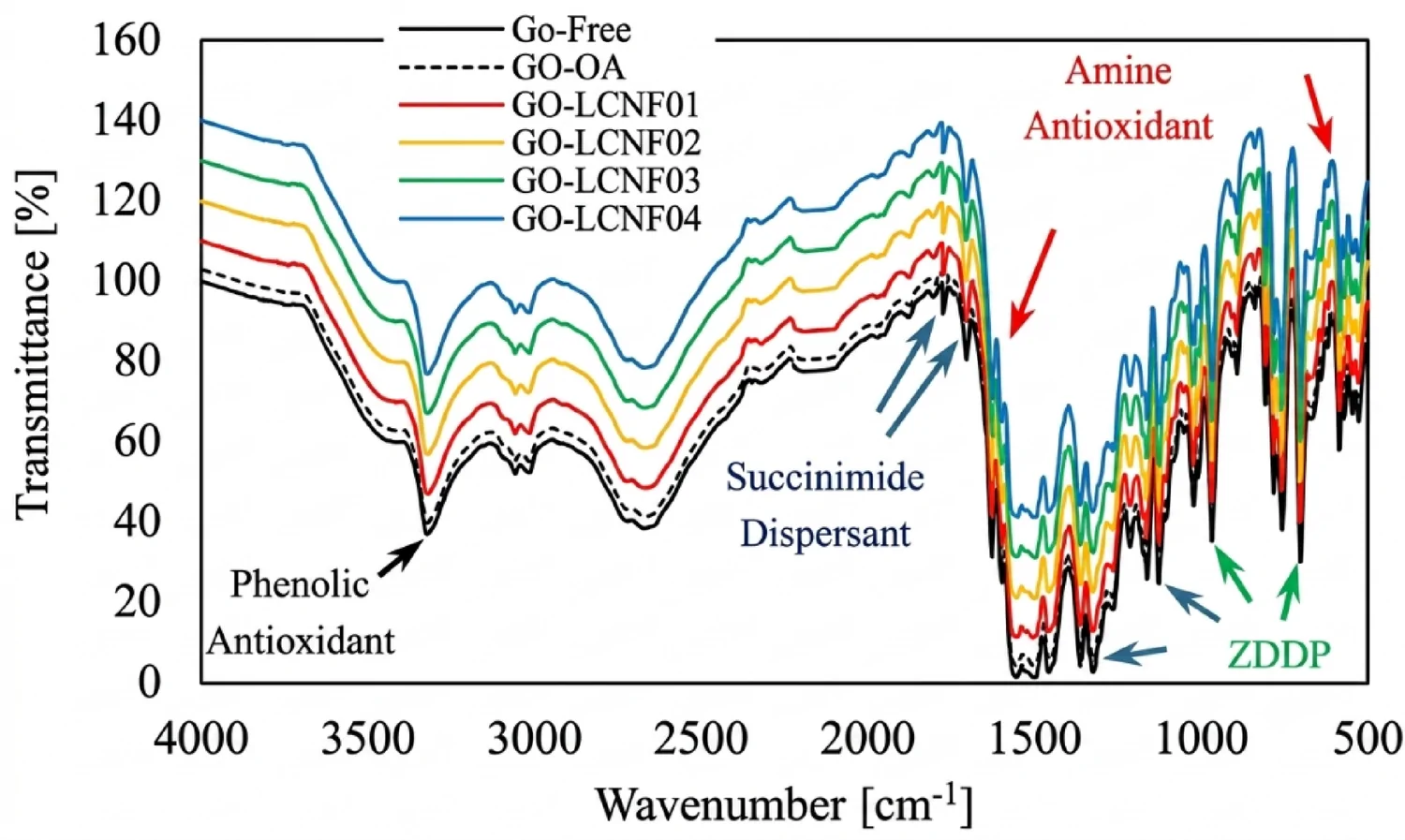
FT-IR wavelength of gear oil dispersed with LCNFs.

The sedimentation observation at three months is depicted in Fig. 3. The results demonstrated that the materials were well mixed and that there was no nanoparticle settling at the test tube’s bottom. Consequently, it was proved that LCNFs nano lubricant remained stable for at least three months. The chemical composition and functional groups of the nanolubricants were analyzed to evaluate their absorption or reflection of the applied infrared radiation. The IR state of the fundamental gear oil exhibits a collection of discrete absorbent bands, as depicted in Fig. 4. The appearance of a peak at about 3600 cm⁻¹ reveals typical vibrations of inhibited phenols, which implies the occurrence of O–H stretching vibrations. It may be noticed that phenolic antioxidants contribute to superior antioxidant stability^50^. The succinimide dispersant also exhibits characteristic C = O stretching and imide-related bending patterns. It can be evident that the appearance of peaks near the regions 1770 cm⁻¹, 1700 cm⁻¹, and 1366 cm⁻¹ confirmed the formation of the dispersant. The thermal and oxidative resistance of the oil develops due to the formation of aromatic amine structures. The generation of amine antioxidants can be confirmed in a unique band near 1500 cm⁻¹, which indicates N–H bending. In addition, the peaks at approximately 650 cm⁻¹ and 980 cm⁻¹ correspond to P–S and P–O–C vibration patterns. These peaks can reveal the presence of zinc dicalcium thiophosphate (ZDDP), which exhibits anti-wear and high-pressure properties^51^.

The identified peaks can serve as reference points for monitoring chemical reactions or structural alterations throughout ingredient dispersion. Moreover, the detected IR wavelength demonstrates a stable and consistent suspension of LCNFs.

**Fig. 5:**
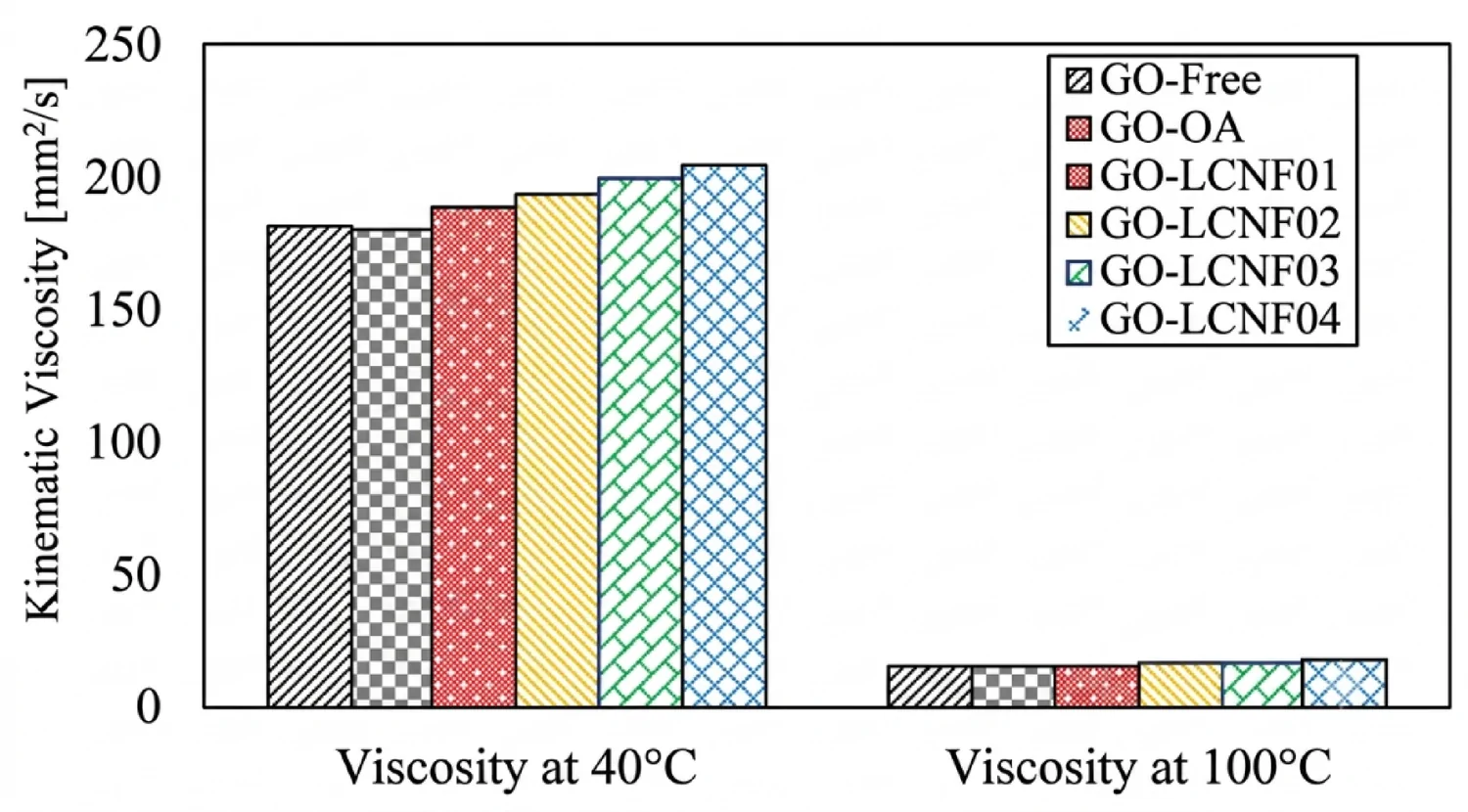
Kinematic viscosity of gear oil dispersed with LCNFs at 40 °C and 100 °C.

**Fig. 6:**
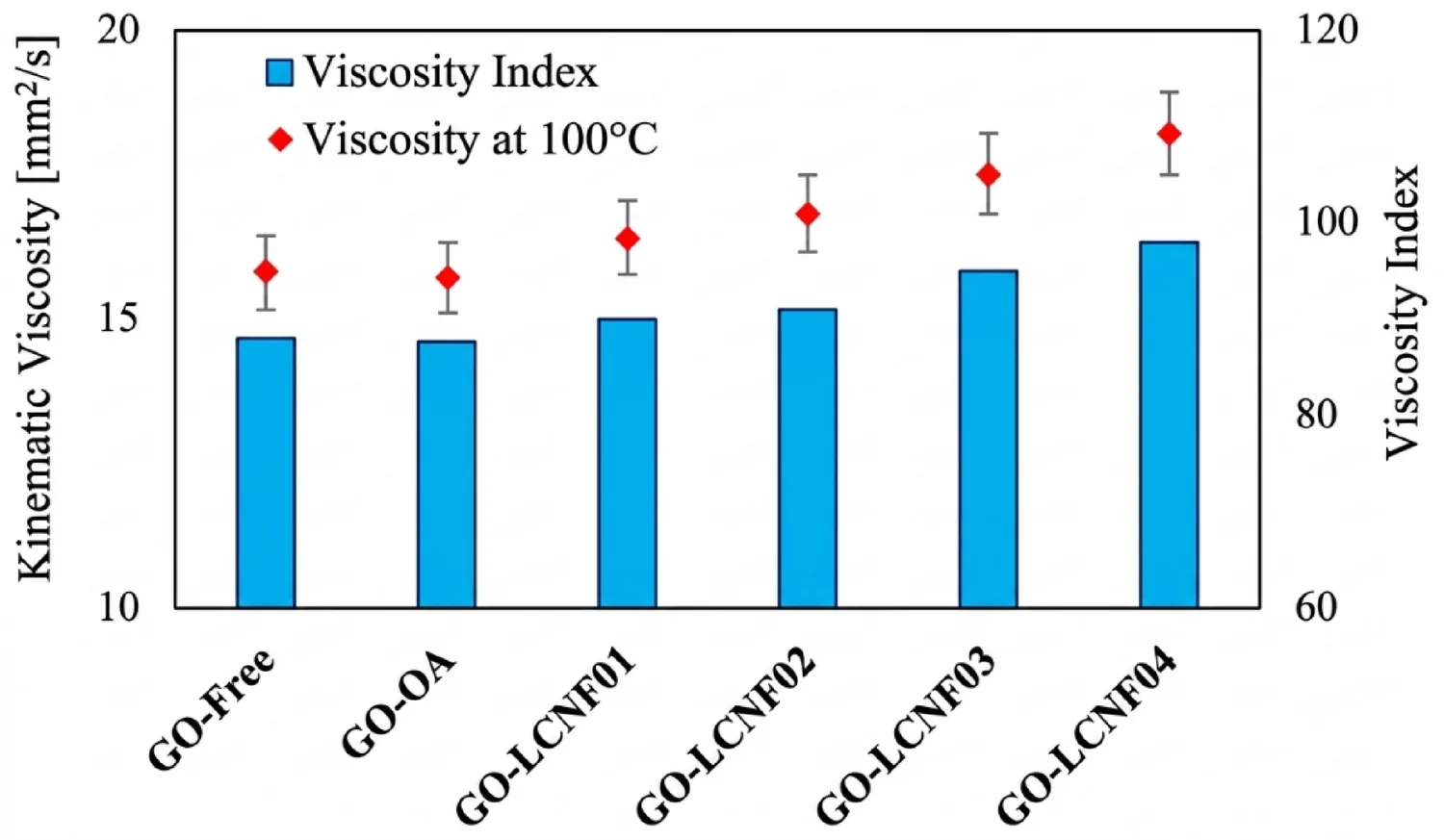
Viscosity Index of gear oil dispersed with LCNFs.

The rheological behavior is significantly impacted by the fibrous composition of LCNFs. Figure 5 depicts the kinematic viscosity of gear oil dispersed with LCNFs at 40 °C and 100 °C. The dynamic viscosity of the GO-Free gear oil sample was measured at 40 °C at 181 mm^2^/s, and a progressive increase in viscosity was observed with increasing LCNF content loading^52^. At a loading level of 0.25% by weight (GO-LCNF01), the viscosity elevated to 189 mm^2^/s, indicating a 4.5% boost. The most significant viscosity value was recorded at a loading amount of 1.0% by weight of LCNFs (GO-LCNF04), wherein the dynamic viscosity approached 205 mm^2^/s, equating to a 13.3% increase relative to the original oil. This may be due to the formation of a thicker and more stable lubricating film, as the fibrous network structure of LCNFs restricts molecular motion^28^. A similar pattern was identified at 100 °C, where GO-Free exhibited a viscosity of 15.8 mm^2^/s. For the GO-LCNF01 sample, the viscosity elevated to 16.4 mm^2^/s (about 3.8% growth), while it obtained 18.2 mm^2^/s with a boost up to 15.2% for the GO-LCNF04 sample. It may also be due to the improved shear resistance of the oil, resulting from the uniform dispersion of LCNFs, which enhances intermolecular interactions. The viscosity index (VI) similarly observed a positive pattern depending on the LCNFs content, as depicted in Fig. 6. The GO-Free sample scored a VI of 88, which elevated to 90 (about 2.2% growth), with the GO-LCNF01 sample. It was found that the highest significant effect of VI was identified at 98 approximately 11.4% growth with the GO-LCNF01 sample. Thus, the growth of the viscosity index demonstrates that the ability of LCNFs to establish a semi-continuous network promotes temperature-dependent rheological durability^53^.

**Fig. 7:**
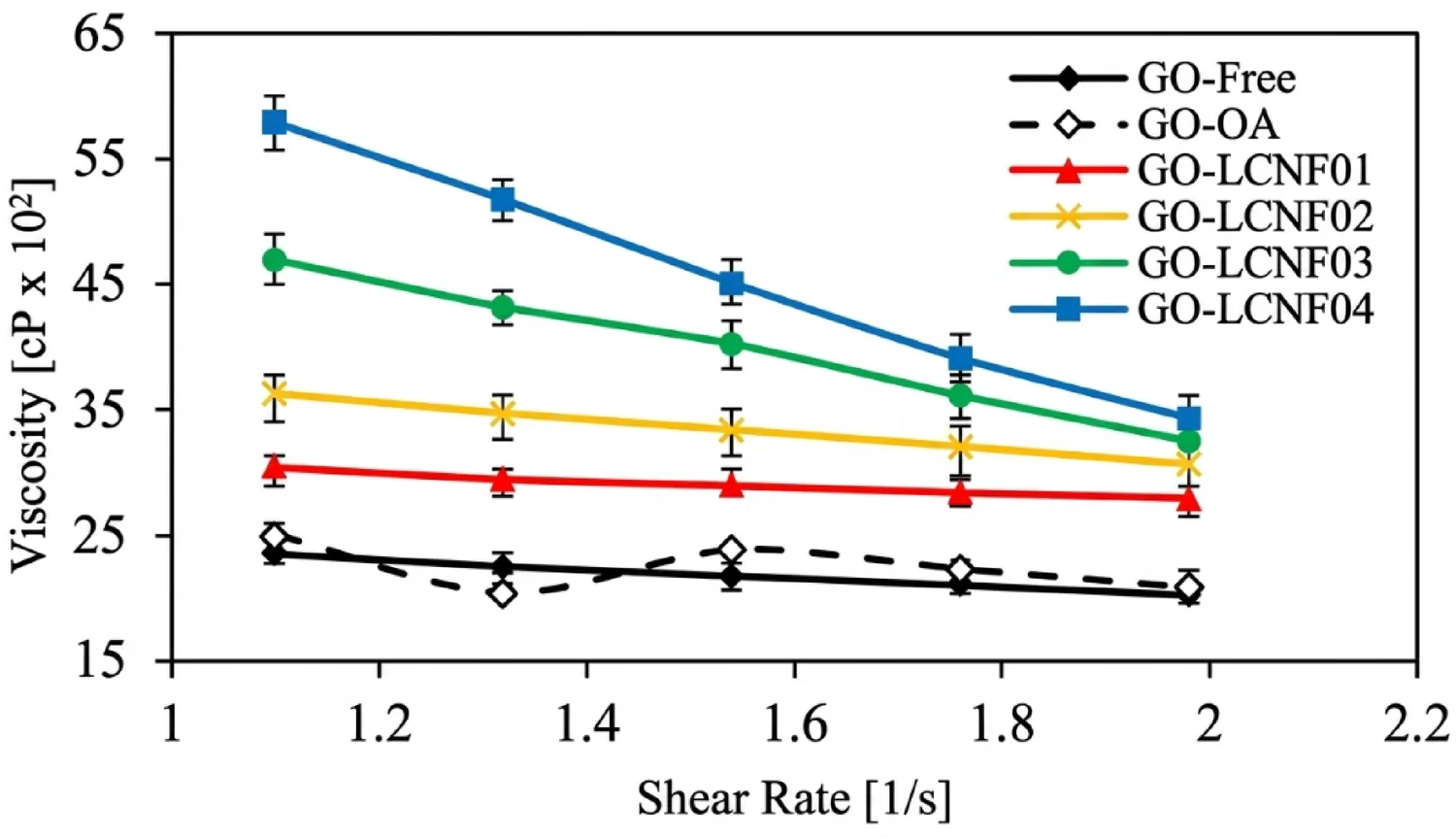
The viscosity Vs shear rate of gear oil dispersed with LCNFs.

The viscosity was plotted against the shear rate at varied concentrations of LCNFs distributed in gear oil, as seen in Fig. 7. It was noticed that the GO-LCNF04 sample exhibited a maximum viscosity over the whole shear-rate range. It was obvious that integration of LCNFs significantly increased the viscosity due to the strong interfacial interaction and network topology. This phenomenon can be due to the high aspect ratio and huge specific surface area of LCNFs. Moreover, the presence of residual lignin inside the LCNF structure promotes greater adhesion and entanglement amongst the scattered nanofibers. This mechanism contributes to the construction of a three-dimensional interconnected network that restricts oil mobility. It can be proved that increasing loading content leads to increased flow resistance, resulting in higher viscosity values.

### Vibration and noise response of nanolubricants

**Fig. 8:**
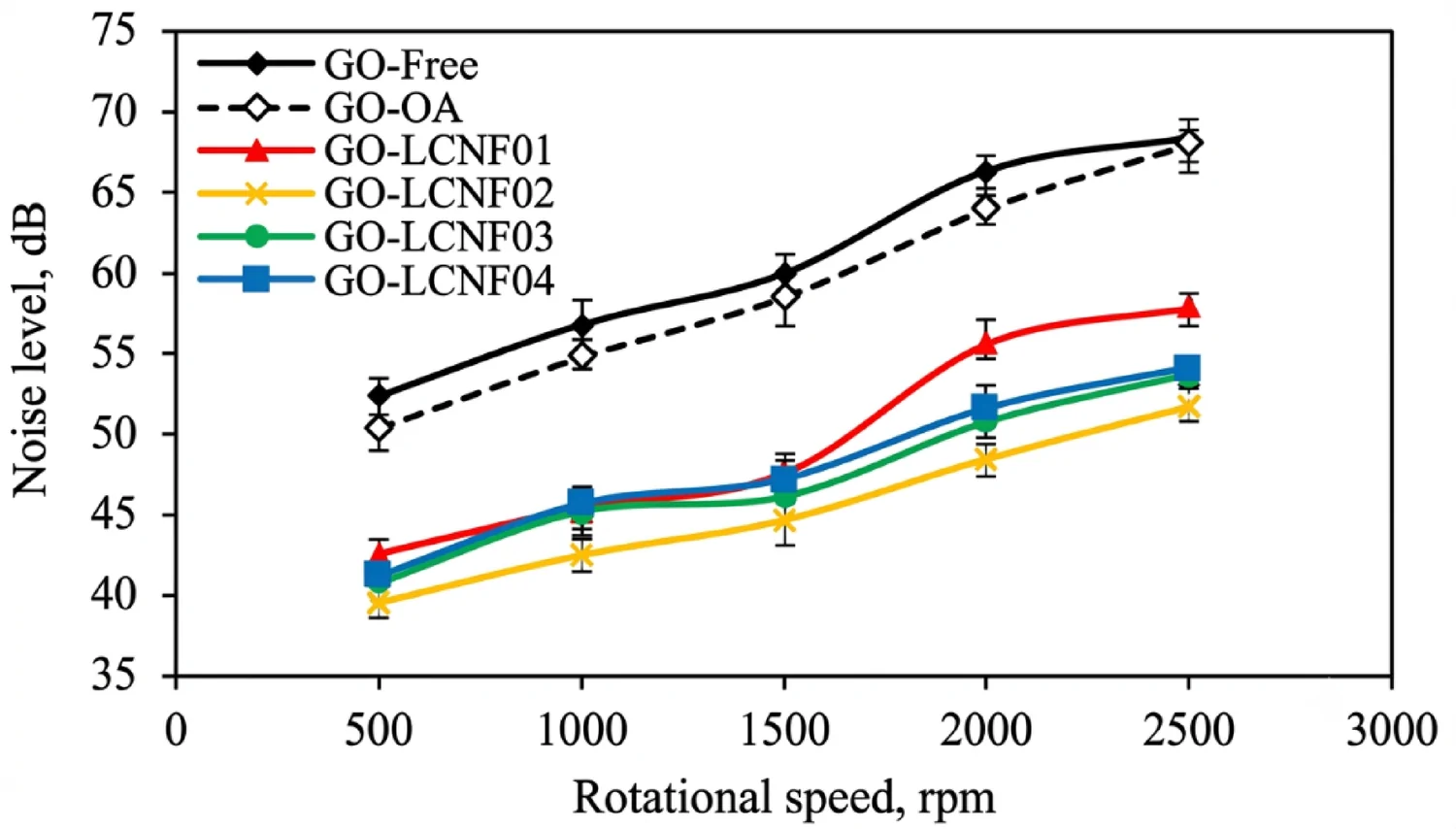
The sound pressure level of gear oil dispersed with LCNFs.

The damping and noise reduction capabilities of nano-lubricants were evaluated by measuring sound pressure level (SPL) and vibration levels. Figure 8 illustrates the sound pressure level of nanolubricants. It was found that SPL decreased noticeably after incorporating LCNFs compared with the free oil additive sample (GO-Free)^54^. It can be indicated that adding a 0.25% loading amount of LCNFs (GO-LCNF01) leads to low SPL up to 18%, demonstrating the positive effect of nanofiber dispersion on noise attenuation. It was evident that loading content of 0.5% by weight (GO-LCNF02) leads to minimizing SPL by approximately 25%, representing the optimal damping behavior. However, increasing the loading above this level led to a slight increase in SPL. It can be indicated that adding a 0.75% loading amount of LCNFs (GO-LCNF03) showed a reduced SPL of 22%, which, although beneficial, is lower than the maximum reduction obtained at 0.5 wt%. The resultant drop is maybe due to LCNFs aggregation at high loading content, which restricts uniform dispersion and restricts the lubricant’s potential to establish a stable damping barrier.

**Fig. 9:**
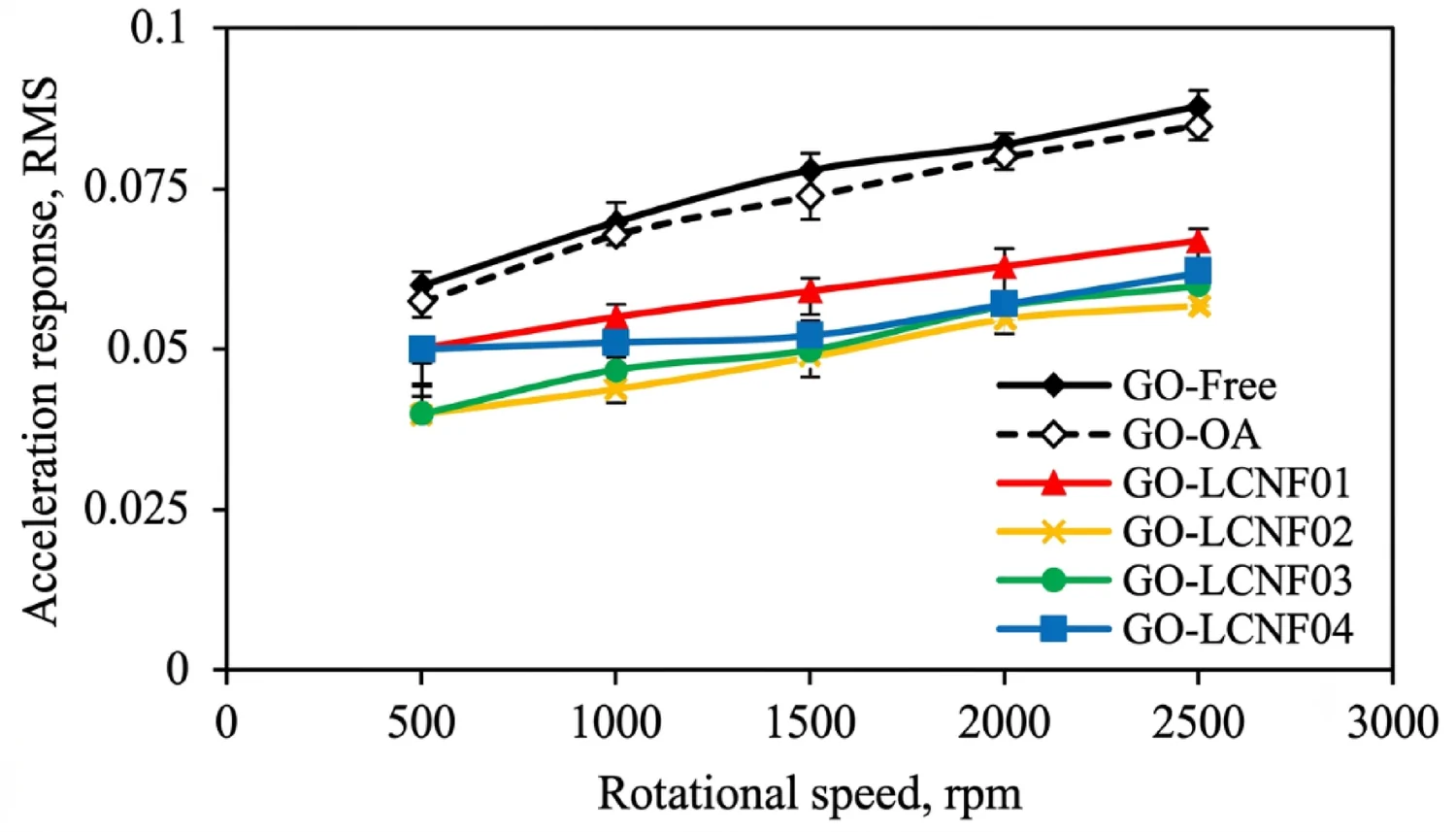
Acceleration response RMS of gear oil dispersed with LCNFs.

A similar trend was observed in the acceleration response (RMS) measurements, as displayed in Fig. 9. It can be indicated that containing 0.25% by weight of LCNFs exhibited an RMS reduction of about 22%. Moreover, it was evident that the 0.5 wt% formulation (GO-LCNF02) achieved the highest reduction of nearly 35%, confirming superior vibration suppression at this concentration. The results reveal that RMS values began to increase again with a high loading amount, above 0.75% by weight. This may be attributed to the aggregation and reduced suspension stability at elevated LCNFs contents. Finally, it can be revealed that LCNFs considerably boost the damping factor and noise-reduction attributes of gear oil^35^. The optimum SPL and vibration (RMS) performance was discovered at a loading level of 0.5% of LCNFs.

### Wear mechanism of nanolubricants

**Fig. 10:**
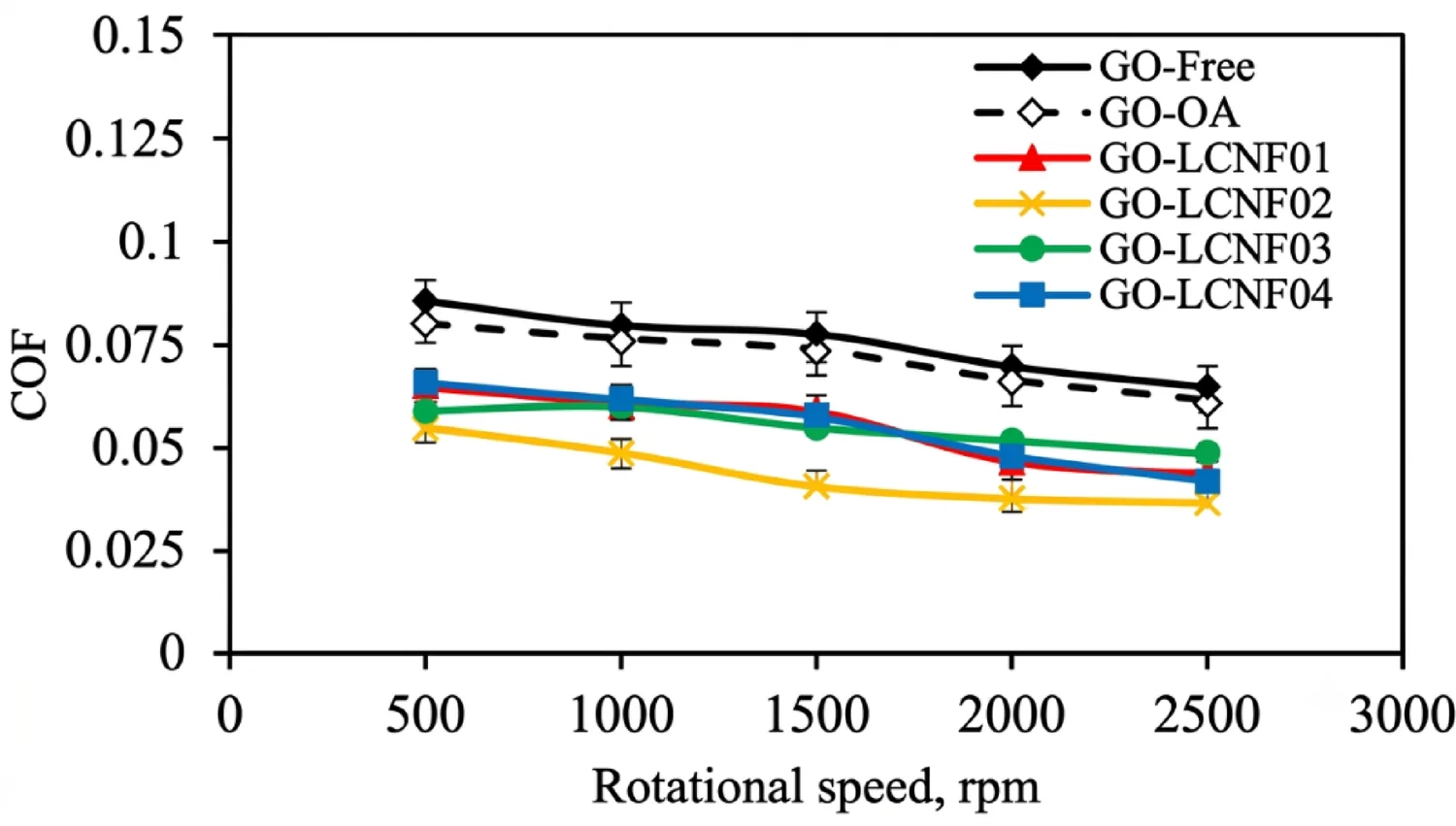
Friction coefficient of gear oil dispersed with LCNFs.

The frictional performance of the LCNFs-dispersed gear-oil samples was assessed and contrasted with the free oil sample. The fluctuation of the friction coefficient (COF) with different rotor speeds is demonstrated in Fig. 10. LCNFs possess surface functional groups that interact well with the base oil components—including oleic acid—improving dispersion uniformity and forming a more stable lubricating layer as the concentration increases^33^. It should be noted that the 2 wt% oleic acid (OA) used in this study primarily functions as a dispersant to improve the stability and dispersion of LCNFs in the lubricant^55,56^. Whereas previous studies have employed substantially higher OA loading content, above 10 wt%, as the primary lubricating additive to directly enhance tribological performance^57,58^. The sample containing 0.25% by weight of LCNFs (GO-LCNF01) showed inferior frictional performance compared to the fresh oil (GO-Free), resulting in approximately a 29% reduction in COF. This initial increase may be attributed to the insufficient amount of LCNFs present to form a continuous boundary lubrication film at the contact interface^59^. With higher LCNF concentrations, the COF decreased noticeably. The GO-LCNF02 sample exhibited superior frictional performance, achieving a 42% reduction in COF. It can be evident that this improved performance may be attributed to the formation of a robust protective nanolayer that reduces asperity interactions. Nevertheless, raising the loading content above 0.5% by weight of LCNFs leads to a drop in efficiency. It may be seen that merely a 27% COF decline was detected in samples with 0.75% and 1.0% by weight of LCNFs, exhibiting low frictional influence. It can be indicated that this degradation may be brought about by inadequate dispersion, stability, and aggregation^60^. This leads to the distortion of layer homogeneity and the prevention of effective boundary lubrication conditions. Therefore, the findings indicate that despite LCNFs greatly enhancing lubrication performance, the ideal content to minimize friction is 0.5% by weight.

**Fig. 11:**
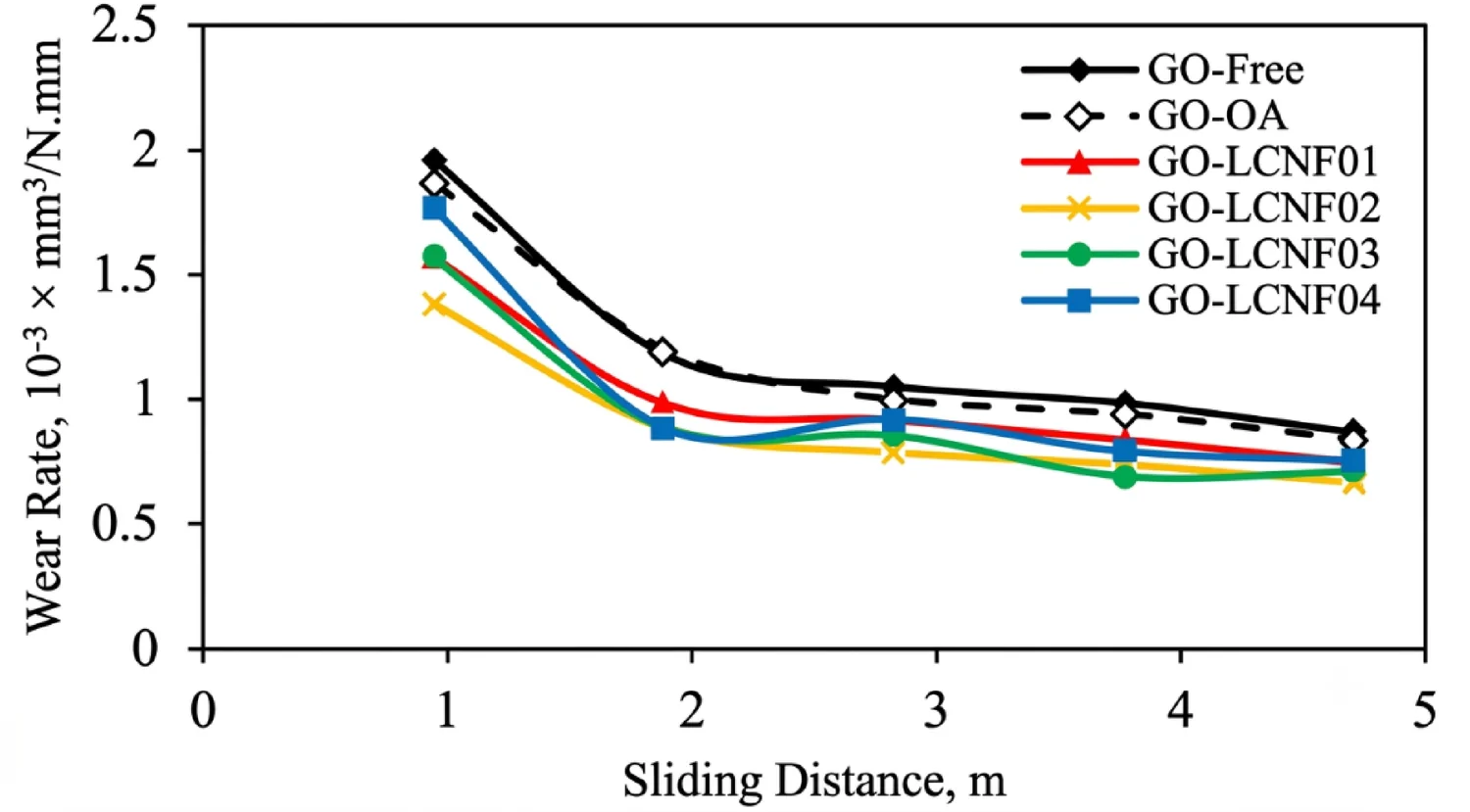
Wear rate of gear oil dispersed with LCNFs.

The wear rates of the LCNFs-dispersed gear oil samples were shown in Fig. 11. It can be noticed that wear resistance was improved with all modified oils, indicating the positive impact of LCNFs. It was found that a loading level of 0.25% by weight LCNFs exhibits a wear-rate reduction of approximately 15%. It may be attributed to LCNFs forming an ultra-thin layer at the mating interfaces. The positive effect continues with increasing the loading level to 0.5% by weight. This can indicate the lowest rate of wear occurring at that concentration (GO-LCNF02), where the wear rate decreased by 25.5% compared with the base oil. It can be concluded that the presence of LCNF in contact areas can reduce abrasive interactions and boost the load-bearing capacity^61^. It also helps mend and fill micro-grooves and asperity valleys, minimizing material loss^62^.

However, it can be shown that increasing the LCNF loading level beyond the optimum resulted in a slight deterioration in yield. Therefore, the samples (GO-LCNF03) and (GO-LCNF04) have recorded high wear compared to the sample (GO-LCNF02). Specifically, the wear rate decreased by 22.3% and 16.2% at loading levels of 0.75% and 1.0% by weight, respectively. It may be attributed to the fact that high LCNF loading increases the agglomeration, leading to unstable dispersion^63^. It also results in non-uniform lubrication and reduced ability to form a surface-protecting film.

**Fig. 12:**
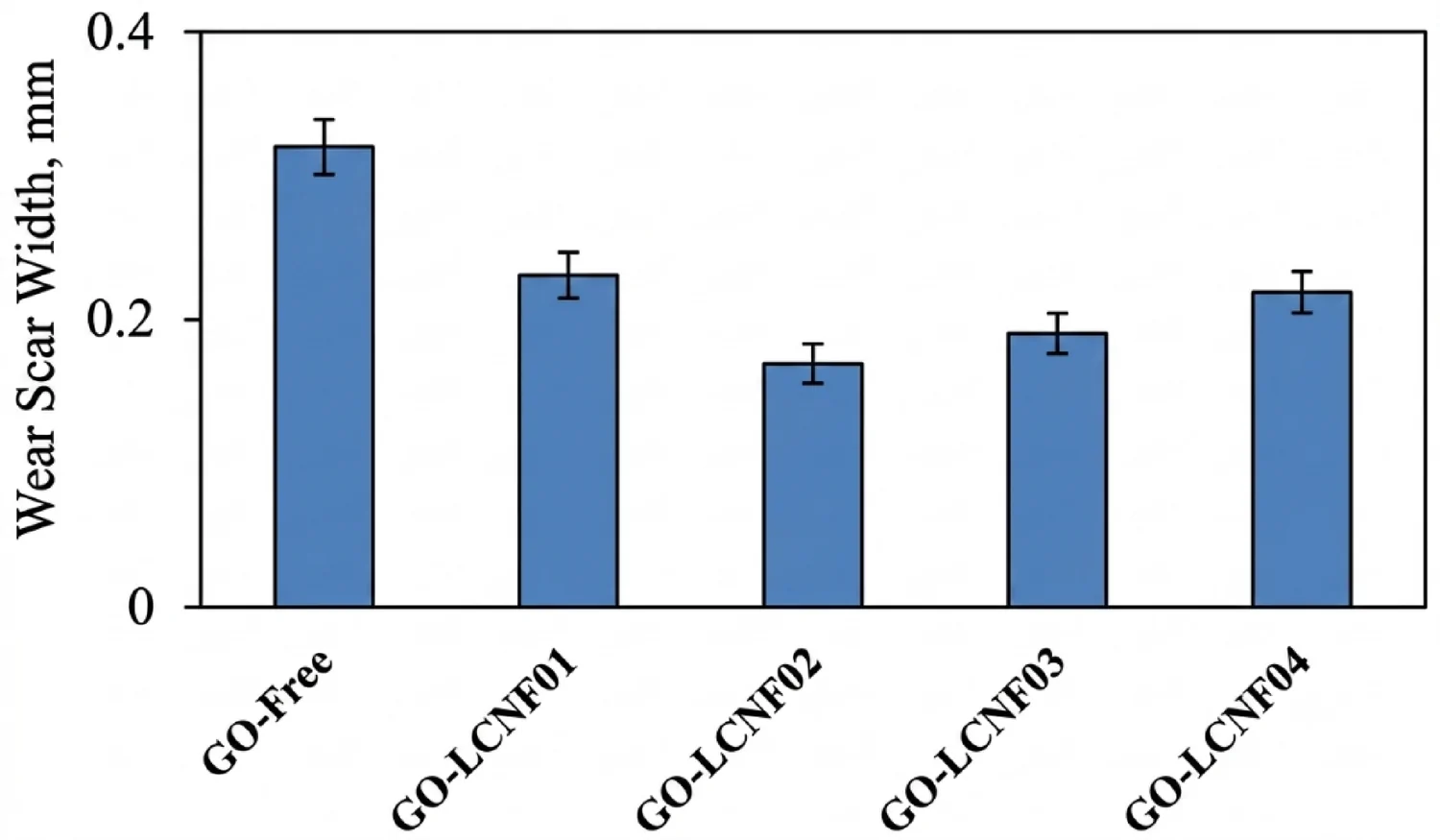
Wear scar width of gear oil dispersed with LCNFs.

For a detailed view of the wear mechanism, the worn surface was evaluated. The wear scar width of sliding surfaces was recorded under 200 N applied load at 2500 rpm rotation speed, as displayed in Fig. 12. It was indicated that the wear scar width of the (GO-Free) sample was 0.32 mm. It can be obvious that the presence of LCNFs contributes to reducing the scar width. The sample (GO-LCNF01) records a scar width of 0.23 mm with reduction of about 28%. Furthermore, the scar width records the highest reduction of 46% with sample (GO-LCNF02). It can be evident that 0.5% by weight of LCNFs is considered optimal, indicating homogeneous dispersion and suspension stability^64^. While it has been shown that high loading content does not help in restricting abrasives, it indicates weak dispersion and increased agglomeration.

**Fig. 13:**
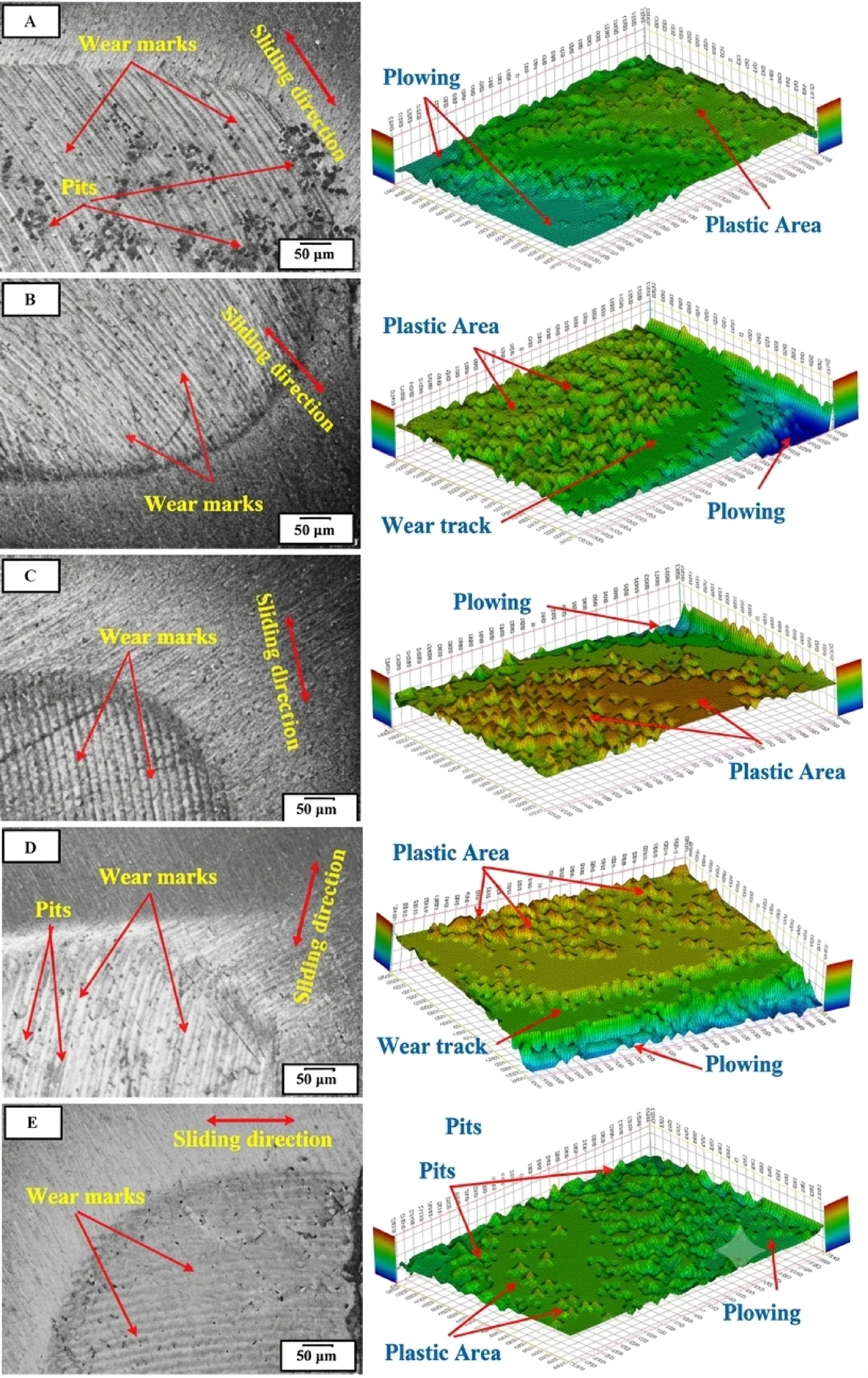
3D topography of worn surface of samples **(a)** fresh sample oil (GO-Free), **(b)** 0.25% LCNFs sample (GO-LCNF01), (c) 0.5% LCNFs sample (GO-LCNF02), (d) 0.75% LCNFs sample (GO-LCNF03) and (e) 1.0% LCNFs sample (GO-LCNF04).

**Fig. 14:**
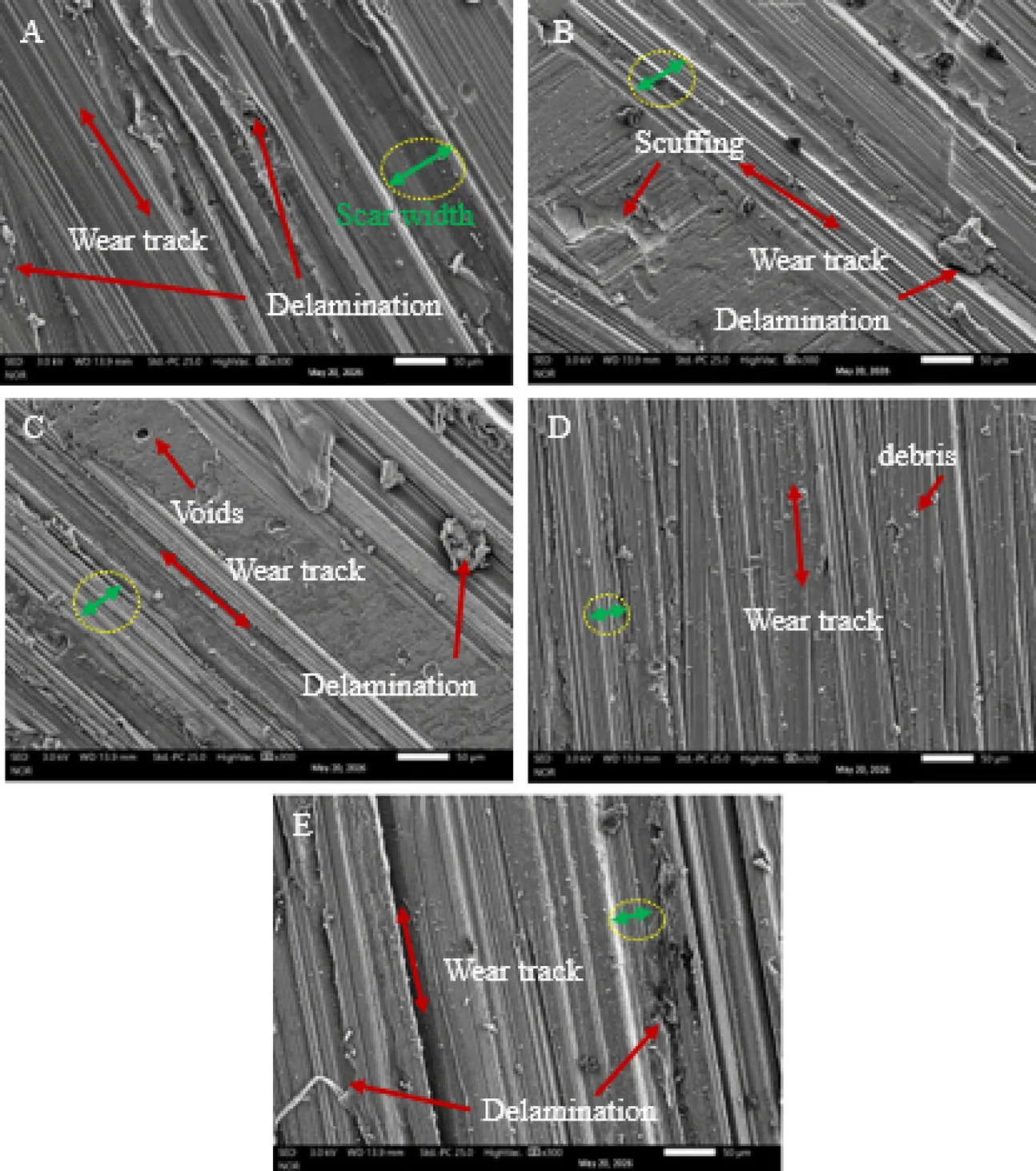
SEM of worn surface of samples **(a)** fresh sample oil (GO-Free), **(b)** 0.25% LCNFs sample (GO-LCNF01), **(c)** 0.5% LCNFs sample (GO-LCNF02), **(d)** 0.75% LCNFs sample (GO-LCNF03) and (e) 1.0% LCNFs sample (GO-LCNF04).

The worn surface characterization was carried out using 3D topography and SEM to understand the wear mechanism under different LCNFs loadings. Figures 13 and 14 depict the 3D topography scan and SEM of worn surfaces of LCNFs samples. It was observed that the surface topography of the sample (GO-Free) reveals a large wear scar with noticeably deep wear tracks. It was noticed that plowing marks and plastic deformation, which may indicate strong abrasive interactions and direct asperity-to-asperity contact^2,33^. The SEM image confirms the presence of peeling and multiple burrs, which refers to abrasive action under boundary lubrication conditions. It can be indicated from the scan surface of the sample (GO-LCNF01) that the wear scar width becomes smaller compared to the fresh oil. The 3D topography of the sample confirms narrow wear tracks with fewer plowing areas^65^. It can be confirmed that low loading content of LCNFs enables effective dispersion and forms a thin protective film. Moreover, SEM images depict fewer wear tracks and burrs. The sample (GO-LCNF02) exhibits the smallest wear scar width and the fewest plowing areas. It can demonstrate that 3D topography shows a semi-uniform surface profile. While the SEM image shows mild plastic wear with minimal scuffing. This demonstrates that the fibrous structure fills microscopic cavities, potentially reducing abrasive wear and minimizing material loss^66^. It was evident that the lignin-based structure provides a boundary lubrication and raises the load-bearing capability. Moreover, LCNFs allow acting as a rolling or sliding buffer, which may directly limit friction and inhibit abrasive reactions. Figure 14d indicates that the wear scar width of sample (GO-LCNF03) begins to increase again. It can be observed that 3D topography reveals increased plowing area and surface irregularities. Therefore, the SEM image indicates an increased spread of burrs^67^. Beyond the loading content of 0.5%, LCNFs begin to agglomerate and tend to form hydrogen bonds. It was confirmed that the agglomeration disrupts the formation of a separating film. A similar behavior to GO-LCNF03 is observed. The scar width and plowing area remain relatively high. 3D topography illustrates plastic deformation and clear wear tracks, while SEM images reveal peeling like the previous sample. It concludes that a load level of 0.5% gives the best frictional performance and the lowest weight loss.

**Fig. 15:**
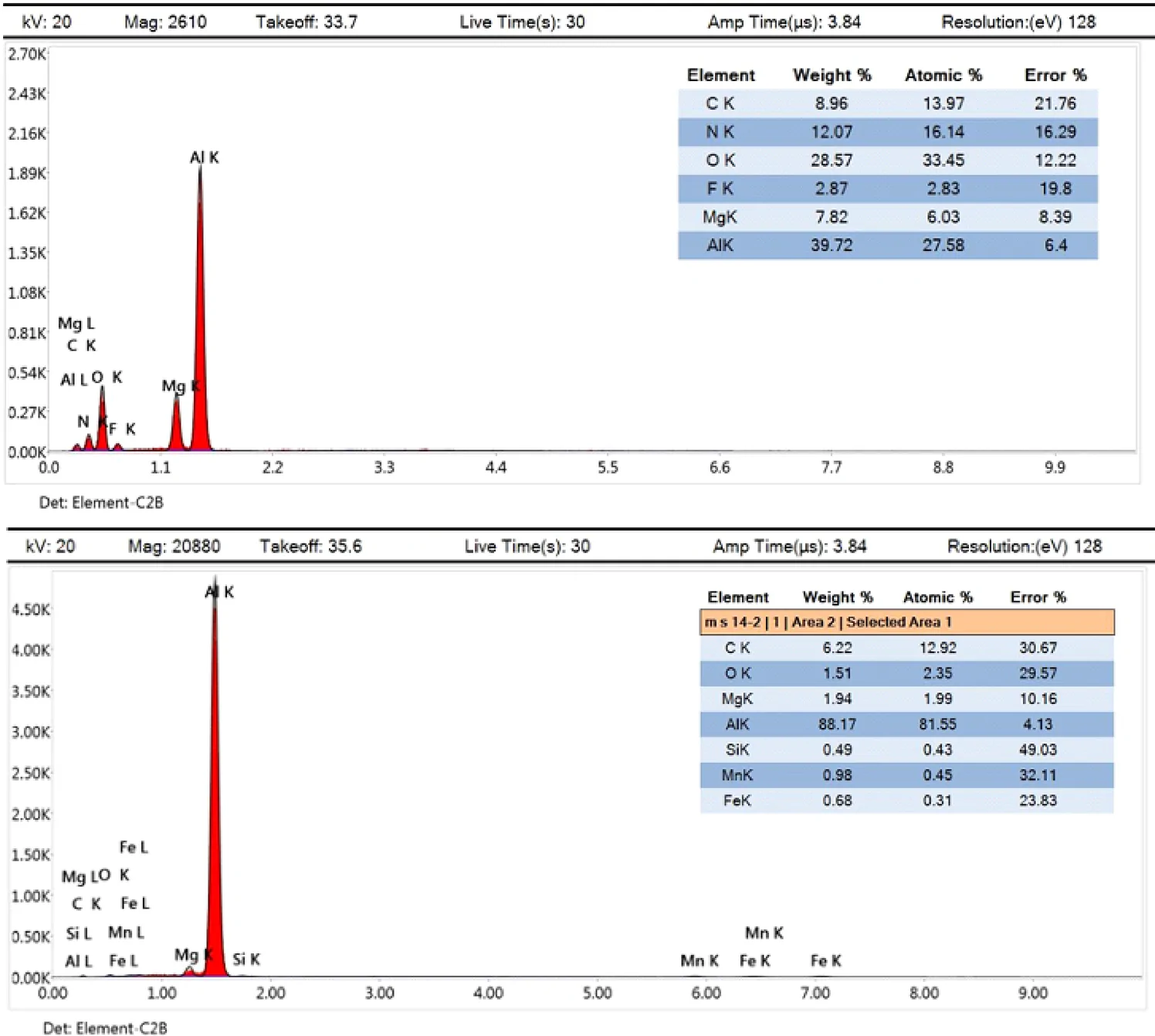
EDX spectrum of plates with non-sliding action and after lubricant sliding contact.

Figure 15 displays the EDS spectra of the plate before and after the sliding operation in wet condition. EDS analysis of the plate before sliding indicates a considerable concentration of oxygen. It was exhibited that the oxygen quantity drastically lowered approximately 2.35 wt% on the worn surface that was lubricated. It may be related to the creation of a protective lubricating layer that shielded the sliding contact from direct exposure to oxygen during the tribological process. It was obvious that the LCNF-based lubricant efficiently prevented surface oxidation and avoided oxide-film accumulation. Furthermore, the sliding action contributed to easing the asperities on the worn surface. It was obvious that the friction process generated a micro-polishing impact on the contact region. The finer surface enabled the EDS analysis to detect the real elemental composition of the substrate material more effectively. As a result, minor levels of Si, Mn, and Fe were discovered combined with Mg in the EDS spectrum. It was claimed these constituents are indicative of structural aluminum alloys. The data verifies the revealing of the underlying alloy matrix after the eradication of unstable oxide layers using traditional sliding.

It can be concluded that what has been accomplished in the current work is promising compared to previous efforts. It can be indicated that the previous studies showed a promising start for a sustainable bio-derived additive. It was revealed that there were significant declines in COF employing CNC-based systems, such as a 69% decrease with 0.1 wt% in SAE40 oil^28^. Moreover, the hybrid CNC–CuO additives exhibit a 48–50% reduction using boundary lubrication conditions^32^. The Hybrid CNCs–Al₂O₃ NPs, with a 2.0 wt% loading level in Bio-gear oil, showed a 35% decrease in wear scar width^35^. In the present work, the LCNF-reinforced gear oil produced a COF reduction of around 42% and wear scar reduction of nearly 46% at an optimum loading level of only 0.5 wt%, indicating comparable tribological performance employing a 100% bio-derived additive solution. In terms of rheological performance, hybrid CNC–CuO additives raised the viscosity index by about 47% and the dynamic viscosity by almost 75% at a loading level of 0.5 wt%^31^. On the other hand, CNCs–Al₂O₃ raised the viscosity index by about 17% at a loading level of 2 wt%^35^. In comparison, the present LCNF system boosted the viscosity index from 88 to 98, equivalent to an enhancement of around 11.4%, while dynamic viscosity increased by nearly 13.3% at 1 wt% loading. Furthermore, the present study indicated additional multifunctional benefits not widely described in prior cellulose-based lubricant investigations. The LCNF-reinforced gear oil reduced vibration and noise signals by around 25–35%, but the hybrid CNCs–Al₂O₃ system reported just an 11% reduction in noise level^35^. It can be concluded that LCNFs provide significant damping behavior and dynamic stability behavior during a sliding scenario.

## Conclusions

The present research emphasizes LCNFs as effective ingredients to enhance the tribological and rheological characteristics of gear oil. Based on rheological experiments, LCNFs elevated dynamic viscosity by approximately 4.5% at a loading content of 0.25% by weight and 13.3% at a loading content of 1.0% by weight. The viscosity index also increased positively with LCNF content, from a base of 88 to a maximum of 98, indicating LCNFs’ role in stabilizing viscosity across temperature changes. Samples modified by LCNFs exhibit superior friction and wear resistance. The results demonstrate that the optimal loading content is 0.5% by weight (GO-LCNF02). It can be revealed that there is significant decrease in wear scar width and COF of approximately 46% and 42%, respectively. It may be attributed to forming a protective tribo-film that boosts load-carrying capacity and inhibits asperity interaction. Excessive loading of content more than 0.5% by weight— led to a moderate decrease in performance due to poor suspension stability and nanoparticle agglomeration.

Furthermore, vibration and noise analysis highlight the positive role of LCNFs in reducing system instability. It can be confirmed that a loading level of 0.5% exhibited the greatest reduction in noise and acceleration signals, ranging from 25% to 35%. It can also be evident that the slip surfaces have become smoother, which enhances damping capabilities. It can be concluded that LCNFs appear to be a very promising and vital component for gear oil. The opportunity to enhance viscosity stability, reduce friction and wear, and improve dynamic response highlights the development of sustainable and highly efficient nano-lubricants. Future studies will focus on direct chemical characterization using XPS and Raman spectroscopy to further confirm the proposed tribofilm formation mechanisms.

## Data Availability

The datasets generated and/or analyzed during the current study are not publicly available but can be obtained from the corresponding author upon reasonable request.
